# Foliar Application of Spermidine Alleviates Waterlogging-Induced Damages to Maize Seedlings by Enhancing Antioxidative Capacity, Modulating Polyamines and Ethylene Biosynthesis

**DOI:** 10.3390/life12111921

**Published:** 2022-11-18

**Authors:** Xiuling Wang, Qun Wang, Moubiao Zhang, Yulong Zhao, Pengfei Dong, Yali Zhao, Hongping Li, Xucun Jia, Panpan An, Yulou Tang, Chaohai Li

**Affiliations:** College of Agronomy, Henan Agricultural University, Zhengzhou 450000, China

**Keywords:** spermidine, abiotic stress, antioxidant defense, polyamine, ethylene biosynthesis

## Abstract

Waterlogging is a major threat to maize production worldwide. The exogenous application of spermidine is well known to enhance plant tolerance to abiotic stresses. The role of exogenous spermidine application in waterlogging tolerance in maize was investigated in this study. Two maize varieties (a waterlogging-tolerant variety: Xundan 20 (XD20) and a waterlogging-sensitive variety: Denghai 662 (DH662)) were subjected to waterlogging stress at the seedling stage, and then foliar spraying of 0.75 mM spermidine or purified water. Findings demonstrated lower chlorophyll content, reduced growth indices, considerable increase in superoxide anion (O_2_^−^) generation rate, and H_2_O_2_/malondialdehyde accumulation in the two maize varieties under waterlogging stress compared to the control treatment. However, the tolerance variety performed better than the sensitive one. Foliar application of spermidine significantly increased antioxidant enzyme activities under waterlogging stress. In addition, the application of spermidine increased polyamine levels and led to the reduction of ethylene levels under waterlogging. Consequences of spermidine application were most apparent for the waterlogging-sensitive cultivar DH662 under waterlogging than the waterlogging-tolerant variety XD20.

## 1. Introduction

Waterlogging is one of the main abiotic stresses that restricts the growth, development, and yield of crops [1]. Maize being the most grown crop worldwide is vulnerable to waterlogging stress, especially at the seedling stage. The precipitation in the Yellow and Huai River valleys in China is mostly concentrated which often causes water accumulation in farmland, resulting in waterlogging of maize. Waterlogging leads to yield losses and hence, it is an important requirement to solve this problem. 

In the areas susceptible to heavy rain and poor ground drainage, flooding has been suggested as a major environmental stress affecting crop production [2]. Waterlogging greatly reduces crop yields and worsens the problem by reducing crop production to feed the growing human population [3]. Waterlogging commonly is caused by heavy rain and depleted soil drainage [4,5]. Higher plants need molecular oxygen for survival and other metabolic processes. However, insufficient supply of oxygen due to the presence of excess water in the soil leads to harmful effects, including the growth inhibition of roots and shoots, along with severe impacts on the phenotype, physiology, and metabolism. Thus, it damages the plants ability to uptake nutrients as well as disturbs the photosynthetic machinery [6]. Furthermore, waterlogging induces lipid peroxidation caused by the excessive accumulation of reactive oxygen species (ROS) [7]. Previous studies have also shown a noticeable reduction in the growth of shoots and roots by higher levels of ethylene [8], and reduced photosynthesis [9,10].

Polyamines (PAs) having a lower molecular weight, are aliphatic nitrogenous compounds, which include putrescine (Put), spermidine (Spd), and spermine (Spm). Amongst them, spermidine has been proven to be involve in strengthening plant tolerance against multiple stresses [11,12]. The catabolism and synthesis of polyamines (PA) have been demonstrated in different plants. Higher plants possess, spermine synthase, arginine decarboxylase, spermidine synthase, S-adenosylmethionine decarboxylase, and ornithine decarboxylase, that are five essential biosynthetic enzymes involved in the biosynthesis of PAs and have already been investigated for many years in a very detailed manner [13]. PAs are usually metabolized by diamine oxidase and PA oxidase. Associations with PAs in plants responding to multiple environmental stresses such as hypoxia [14], salt stress [15], drought [12], cold temperatures [16], and others [17], as a result of their involvements in the stability of membrane, free radical scavenging while maintaining nucleic acids and the protein structures [18] have been well-documented. Li et al. [19] found that root application of spermidine can significantly improve the growth of cucumber seedlings, by regulating PA levels and root respiration under hypoxia. However, the underlying mechanisms of Spd-induced waterlogging tolerance in maize are not yet been entirely understood.

The goal of this research was to elucidate the underlying mechanisms of Spd-promoting maize waterlogging tolerance through leaf spraying. The experiments could deliver key scientific information to deeply understand the mechanism of Spd in the promotion of maize waterlogging tolerance.

## 2. Materials and Methods

### 2.1. Plant Materials and Stress Treatment

Seeds of two maize cultivars (waterlogging-tolerant XD20 and waterlogging-sensitive DH662) were sown into pots (6 cm down-diameter, 8 cm up-diameter, 12 cm deep) that contained fine soil and the pots were placed in plastic tubs (60 cm × 40 cm × 15 cm). The soil particle constituents and content were: <0.002 mm, 14%; 0.002–2 mm, 29%; 0.02–2 mm, 57%; organic matter, 7.74 g·kg^−1^; available nitrogen, 30.77 mg·kg^−1^; available phosphorus, 11.84 mg·kg^−1^; available potassium, 95.24 mg·kg^−1^. After germination seedlings at the 3-leaf-1-heart stage were divided into three sub-groups (i) control (CK), (ii) waterlogging (W) and (iii) W+ spermidine (WS) treatment. For the control group, plants were irrigated normally. For waterlogging treatment, the water level was kept 2 cm above the soil surface. Spermidine (0.75 mM) dissolved in water was sprayed two times a day at 7 am and 7 pm, respectively. Seedlings of control (CK) and waterlogged (W) groups were sprayed with an equal volume of distilled water (200 mL). This pot experiment was conducted at the Henan Agricultural University in a glasshouse under natural light with an average daily temperature of 25–28 °C and a “12 h/12 h” photoperiod. The light intensity was maintained at 1000 µmol m^−2^ s^−1^, whereas a relative humidity of 60 ± 5% = was recorded. Leaf samples were collected after three days of treatment for subsequent analysis.

### 2.2. Measurement of Chlorophyll Content and Other Growth Attributes

For the assessment of the combined effects of exogenous Spd and waterlogging in maize seedlings, chlorophyll content along with other growth attributes, such as plant height, shoot and root fresh weight were recorded. The first leaves of four plants per group were used for chlorophyll measurements by using a SPAD-502 chlorophyll meter (Minolta Co., Ltd., Osaka, Japan). Shoot and root fresh weights were measured by using a Hc-B085 Laboratory Electric Balance.

### 2.3. Measurement of Reactive Oxygen Species (ROS) and Malondialdehyde (MDA) Contents

The O_2_^−^ production rate was determined by using the hydroxylamine oxidation method as described by [20]. The H_2_O_2_ was measured using the kit YX-W-A400 (Sino Best Biological Technology Co., Ltd., Beijing, China). MDA content was estimated following the method described by [21].

### 2.4. Protein Extraction and Estimation of Antioxidant Enzyme Activities

For the extraction of protein, the leaf samples (0.1 g) were homogenized in a phosphate-buffered solution in an ice bath. The crude extracts were centrifuged for 20 min at 15,000× *g* before the estimation of antioxidant enzyme activities. The protein content was measured according to Bradford [22]. The standard used was BSA. Superoxide dismutase (SOD) activity was determined by using the nitroblue tetrazolium photoreduction method as described earlier by [23]. Peroxidase (POD) activity was measured by using the guaiacol method reported earlier by [24]. Additionally, catalase (CAT) activity was measured according to the procedure described by Shang et al. [25].

The enzymatic activity of ascorbate peroxidase (APX), glutathione reductase (GR), monodehydroascorbate reductase (MDHAR) and dehydroascorbate reductase (DHAR) was measured by using the kit YX-W-A304, kit YX-W-A111, kit YX-C-A008, and kit YX-C-A306 (Sino Best Biological Technology Co., Ltd.), respectively.

### 2.5. Estimation of Non-Enzymatic Antioxidants

Leaf samples (1 g) were ground in cold meta-phosphoric acid (5%). After grinding, the samples were subjected to centrifugation at 11,500 RPM for 15 min at 4 °C. The clear supernatant was collected for the analysis of ascorbate and glutathione content. Ascorbic acid + dehydrogenases activity (AsA + DHA) and ascorbic acid (AsA) content were analyzed according to the protocol of Zhang et al. [26]. The concentration of dehydrogenases activity (DHA) was estimated by the difference between AsA + DHA and AsA. Levels of oxidized glutathione (GSSG) along with total glutathione (GSH + GSSG) were estimated following the method of Nahar et al. [27]. Contents of GSH were computed by deducting GSSG from the total GSH.

### 2.6. Determination of Polyamines

Content of polyamines (PAs) was measured by high-performance liquid chromatography as described by [28]. The leaf samples (0.2 g) were ground and homogenized with 5 mL of 5% pre-cooled perchloric acid. The samples were then left standing at 4 °C for an hour, and then centrifuged at 17,000× *g* for 30 min. The supernatant (0.5 mL) was collected, 1 mL of 2 mol/L NaOH was added to it. Then, 10 µL benzoyl chloride was added, vortexed, and placed in a water bath at 37 °C for 30 min. Saturated NaCl (2 mL) as well as ether (3 mL) were added followed by centrifugation for 5 min at 500× *g*. A volume of 1.5 mL of ether phase was collected into a centrifuge tube and evaporated till dry using hot air. Next, 100 µL of 60% (*v*/*v*) methanol was added to dissolve, put on a 0.45 µm filter membrane, and 10 µL was taken for injection (the same method was used to prepare the putrescine, spermidine and spermine standards). The eluent used was 64% methanol (*v*/*v*), the flow rate was 0.7 mL/min, and the column temperature was 25 ± 1 °C. Readings were taken at the wavelength of 254 nm with a Waters Model 2487 UV detector. PA content is expressed in nmol·g^−1^ fresh weight (nmol·g^−1^ FW).

### 2.7. Measurement of Polyamines Metabolic Enzymes Activities

The determination of arginine decarboxylase (ADC), ornithine decarboxylase (ODC), and S-adenosylmethionine decarboxylase (SAMDC) was performed as described by Zhao et al. [29] and Hu et al. [30]. Activities of polyamine oxidase (PAO) and diamine oxidase (DAO) were monitored as described by Hu et al. [30].

### 2.8. Quantification of Ethylene

The method already described by Yamauchi et al. was followed for ethylene measurements [31]. The leaves were taken from maize seedlings and then placed in a container containing a solution of saturated sodium chloride. The gas excreted from the maize seedlings under vacuum conditions was gathered in a test tube via a funnel. Gas samples (1 mL) were removed from the vial head space with a high-precision air-tight syringe and manually injected into gas chromatography (GC-2010, Shimadzu Co., Kyoto, Japan). Four biological replicates were taken for the ethylene measurements.

### 2.9. Extraction of RNA and Quantitative Real-Time PCR Analyses

Frozen leaves were used for the extraction of total RNA by the Trelief^TM^ RNAprep Pure Plant Kit (TSINGKE, Beijing, China) as per the instructions given by manufacturer. To perform the qRT-PCR, 1 µg from the total RNA was used as a template. Transcript concentrations were quantified using a StepOnePlus Real-Time PCR System (Applied Biosystems, Foster City, CA, USA) and ChamQ Universal SYBR qPCR Master Mix (Vazyme Biotech Co., Ltd., Nanjing, China) as mentioned by Yamauchi et al. [31]. As an internal control actin 7 was used. Three biological replicates were used for qRT-PCR. The primer sequences are presented in Supplementary Table S2.

### 2.10. Statistical Analyses

Data are stated as mean ± standard deviation (SD) of three or four independent repeats. The analysis of data along with graphical presentations were performed in Microsoft Excel 2007, SPSS 19 (SPSS Inc., Chicago, IL, USA). According to the Bartlett test, 6/78 samples with *p* values less than 0.05 were heterogeneous, and 72/78 samples were homogeneous. At the same time, 4/78 samples with *p* values less than 0.05 did not show normal distribution, and 74/78 samples showed normal distribution (Supplementary Table S1).

## 3. Results

### 3.1. Variations in Chlorophyll Content and Growth Attributes

**Figure 1:**
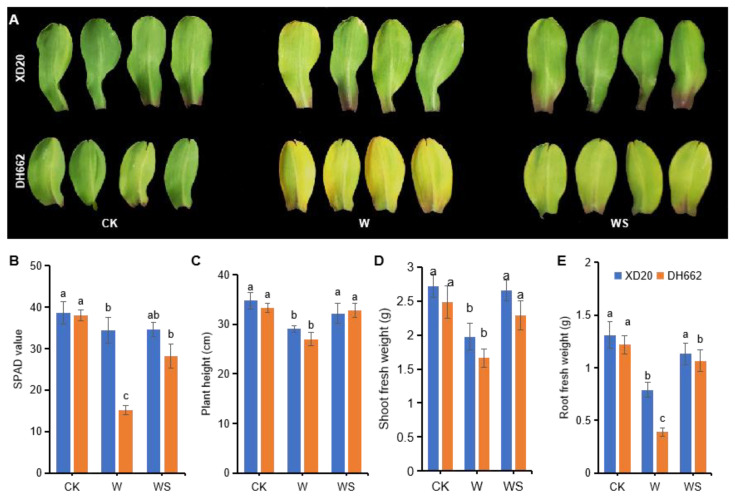
Impacts of spermidine on maize plants in waterlogging stress. The representative figure (**A**), SPAD values (**B**), plant height (**C**), fresh weight of shoots (**D**), and fresh weight of roots (**E**) of 10-day-old plants of the maize cultivars XD20 and DH662 subjected to waterlogging stress and then spraying with 0.75 mM spermidine twice a day for 5 days. Vertical bars show mean values ± SD (*n* = 4). Different lower-case letters above each bar represent significant differences at *p* < 0.05 (LSD test). Where CK, W and WS represent, control, waterlogging and waterlogging stress plus spermidine spraying, respectively.

To determine the ability of spermidine to overcome the impact of waterlogging stress and improve maize plant growth, the chlorophyll content and plant growth parameters were analyzed and compared in tolerant and sensitive cultivars. The SPAD values of the tolerant XD20 cultivar, showed no significant differences in response to waterlogging (W) or waterlogging plus spermidine (WS) treatment. While in DH662 (sensitive cultivar), the SPAD values of waterlogged plants were significantly decreased by 60.13% compared to the control (CK), (Figure 1A,B), whereas spermidine application enhanced the SPAD values by 86.47%. Under waterlogging conditions, a significant reduction in plant height and shoot fresh weight was observed compared to CK in both cultivars (Figure 1C–E). Upon spermidine application, plant height and shoot and root fresh weight showed a significant increase in DH662 (21.26, 37.84, and 172.64%), compared to XD20A (10.83, 34.64, and 43.02%) under waterlogging (W) stress. Hence, DH662 was more sensitive to spermidine application than XD20 and exogenous spermidine application under waterlogging stress led to an increase in chlorophyll content, plant height, and shoot and root fresh weight.

### 3.2. Impacts of Exogenous Spd on O_2_^−^ Production, H_2_O_2_, and MDA Content

**Figure 2:**
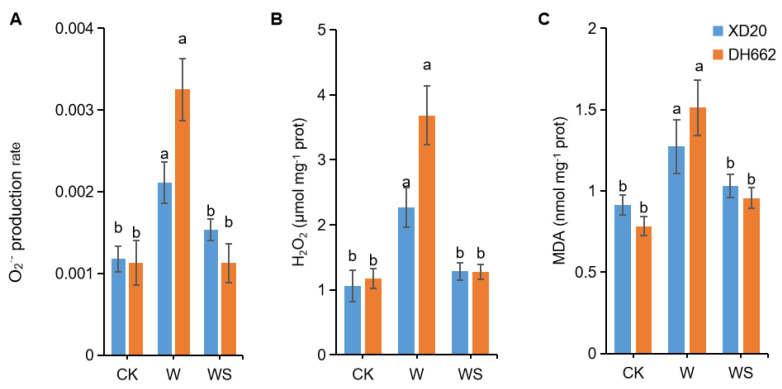
Effects of spermidine on the O_2_^−^ generating rate (**A**), H_2_O_2_ (**B**), and MDA (**C**) in leaves of maize subjected to waterlogging. Control (CK) and waterlogged (W) plants were sprayed with distilled water, while WS plants were sprayed with 0.75 mM spermidine. Data are expressed as mean ± SD of four replications. Different lower-case letters above bars show significant differences at *p* < 0.05 (LSD test).

The effect of spermidine on innate plant tolerance against waterlogging stress was determined by measuring the changes in O_2_^−^, H_2_O_2_ and MDA content. For both maize cultivars, a significant increase of 79.14, 113.96, and 39.30% in XD20 and in the case of DH662 an increase of 187.22, 214.64, and 92.97%, respectively, was observed for the O_2_^−^ production rate, H_2_O_2,_ and MDA content under waterlogging relative to CK (Figure 2A–C). In comparison to waterlogging stress, spermidine application (WS) in XD20 and DH662 led to a significant decrease in the O_2_^−^ production rate, H_2_O_2,_ and MDA content by 27.08, 43.41, 18.89% and 65.36, 65.32, 36.74%, respectively. The effect of waterlogging on reactive oxygen species (ROS) levels in the leaves of DH662 was greater than those of XD20, and the positive regulatory effect of spermidine was also more significant in DH662.

### 3.3. Variations in the Activities of SOD, POD, and CAT

**Figure 3:**
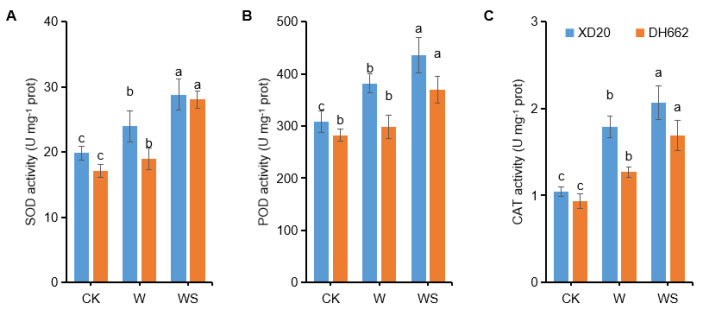
Antioxidant enzyme activities in response to exogenous spermidine. SOD (**A**), POD (**B**), and CAT (**C**). Control (CK) and waterlogged (W) plants were sprayed with distilled water, while WS plants were sprayed with 0.75 mM spermidine. Data are expressed as mean ± SD of four replications. Different lower-case letters above bars show marked differences at *p* < 0.05 (LSD test).

To eliminate the harmful effects of elevated levels of ROS in stressed plants, antioxidant enzymes are produced to scavenge these ROS. Therefore, the production of antioxidant enzymes such as SOD, POD and CAT were analyzed under waterlogging stress in sensitive and tolerant cultivars. A marked increase in the activities of SOD, POD, and CAT by 20.89, 23.57, and 71.07%, respectively, was observed in XD20 compared to CK (Figure 3A–C). For DH662, only the CAT activity was significantly increased under waterlogging by 35.66%. Compared with waterlogging stress alone, the exogenous spermidine further enhanced the activities of SOD, POD, and CAT by 20.28, 14.21, and 15.59%, respectively in XD20, whereas an increase of 47.67, 23.43, and 33.11%, respectively was observed in DH662. Hence, it can be concluded that exogenous spermidine application can enhance antioxidant enzyme activities to reduce elevated levels of ROS, thus increasing maize plant tolerance to waterlogging stress.

### 3.4. Variations in the Activities of APX, GR, MDHAR, and DHAR

**Figure 4:**
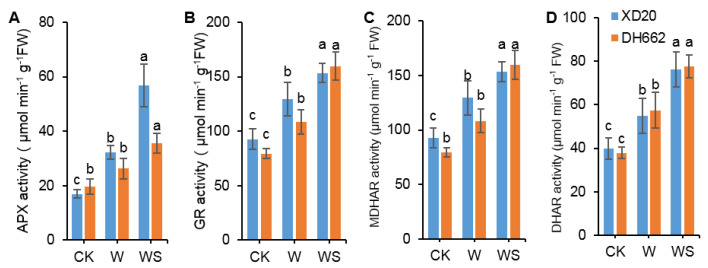
Variation in the activities of APX (**A**), GR (**B**), MDHAR (**C**), and DHAR (**D**). Control (CK) and waterlogged (W) plants were sprayed with distilled water, while WS plants were sprayed with 0.75 mM spermidine. Data are expressed as mean ± SD of four replications. Different lower-case letters above bars indicate significant difference at *p* < 0.05 (LSD test).

The role of spermidine in enhancing waterlogging tolerance was determined in sensitive and tolerant maize cultivars by examining the activities of different enzymes. Current results indicate that under waterlogging stress, APX, GR, MDHAR, and DHAR activities in XD20, were enhanced by 89.64, 39.66, 54.03, and 37.51%, respectively compared to CK. Whereas in DH662, an increase of 36.54 and 51.68%, respectively was observed in GR and DHAR activities (Figure 4A–D). Spermidine application enhanced the activities of APX, GR, MDHAR, and DHAR by 76.43, 18.57, 27.77, and 38.84%, respectively in XD20, and 35.28, 47.34, 51.19, and 35.09%, respectively in DH662. Hence, our results indicate that spermidine application can increase the activities of APX, GR, MDHAR, and DHAR to enhance waterlogging tolerance in maize.

### 3.5. Effects of Exogenous Spd on the Non-Enzymatic Antioxidants

**Figure 5:**
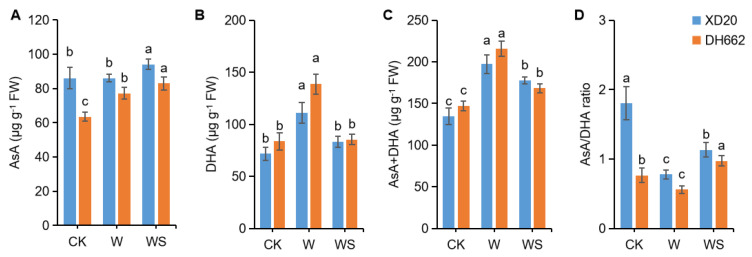
Effects of exogenous spermidine application on non-enzymatic antioxidants, AsA (**A**), DHA (**B**), AsA + DHA (**C**), and AsA/DHA ratio (**D**). Control (CK) and waterlogged (W) plants were sprayed with distilled water, while WS plants were sprayed with 0.75 mM spermidine. Data are expressed as mean ± SD of four replications. Different lower-case letters above bars show significant difference at *p* < 0.05 (LSD test).

AsA and DHA play a vital role in ROS scavenging in plants. In order to establish the role of non-enzymatic antioxidants in developing tolerance to waterlogging stress in maize upon spermidine application, the activities of AsA, AsA + DHA and AsA/DHA were measured. In DH662, a 21.54% increase in the level of AsA was observed, but no increase was observed in XD20 compared to CK. Upon spermidine application, the AsA content in XD20 and DH662 increased by 9.39 and 7.37%, respectively (Figure 5A). A significant increase in the levels of DHA (130.53 and 65.35%) and AsA+DHA (46.82 and 46.45%) was observed; however, a significant reduction in the AsA/DHA ratio was observed in XD20 and DH662 compared to the control (Figure 5B–D). Upon exogenous spermidine application a significant decrease in DHA as well as AsA + DHA was observed in comparison to the seedlings subjected to waterlogging only. However, a significant increase in the AsA/DHA ratio by 45.55 and 73.75% was observed in XD20 and DH662, respectively. Overall, it can be assumed that the higher activities of these non-enzymatic antioxidants could lead to a better waterlogging tolerance by applying exogenous spermidine.

**Figure 6:**
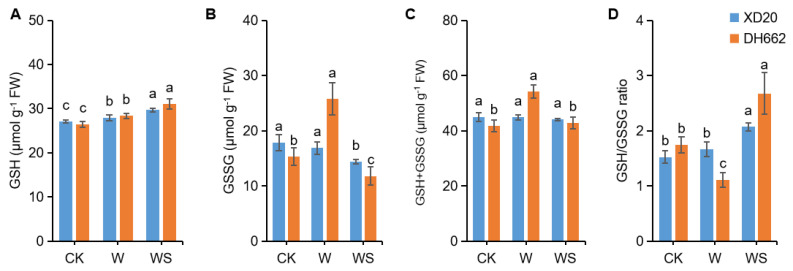
Impacts of Spd on the content of GSH (**A**), GSSG (**B**), GSH + GSSG (**C**), and GSH/GSSG ratio (**D**). Control (CK) and waterlogged (W) plants were sprayed with distilled water, while WS plants were sprayed with 0.75 mM Spd. Data are expressed as mean ± SD of four replications. Different lower-case letters above bars show significant differences at *p* < 0.05 (LSD test).

ROS scavengers such as GSH and GSSG are crucial in enhancing biotic or abiotic stress tolerance in plants. Herein, the activities of GSH and GSSG were measured in response to exogenous spermidine application under waterlogging stress to determine their potential roles in enhancing waterlogging tolerance. An increase of 3.29 and 7.11% in GSH level in XD20 and DH662, respectively, was observed. Current results exhibited an increase of 68.73% in GSSG levels in DH662 only compared to the control under waterlogging stress. Exogenous spermidine application led to an increased in GSH levels (6.33 and 9.68%) but decreased GSSG content (14.78 and 54.24%) in XD20 and DH662, compared to the seedlings subjected to the waterlogging alone (Figure 6A,B). An increase of GSH + GSSG content was observed in DH662 in response to waterlogging, and spermidine application decreased the GSH + GSSG content (Figure 6C). Waterlogging exerted no significant effects on XD20, but a decrease in the GSH/GSSG ratio was observed in the case of DH662 (Figure 6D). Exogenous spermidine application remarkably increased the GSH/GSSG ratio in XD20 and DH662 by 24.37 and 140.82%, respectively, thus making maize seedlings more tolerant to waterlogging.

### 3.6. Effects of Exogenous Spd on Endogenous Put, Spd, and Spm

**Figure 7:**
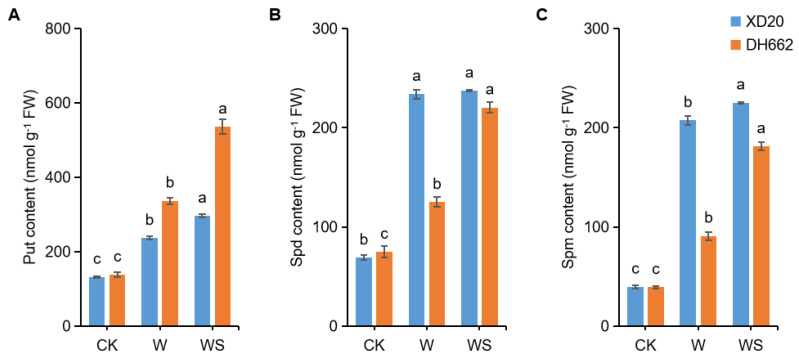
Effects of exogenous spermidine on Putrescine (Put, (**A**)), Spermidine (Spd, (**B**)) and Spermine (Spm) (**C**)). Control (CK) and waterlogged (W) plants were sprayed with distilled water, while WS plants were sprayed with 0.75 mM spermidine. Data are expressed as mean ± SD of four replications. Different lower-case letters above bars demonstrate a significant difference at *p* < 0.05 (LSD test).

Endogenous putrescine, spermidine, and spermine participate in tolerance to various biotic and abiotic stresses in plants. To estimate the impacts of spermidine application on the activities and formation of endogenous putrescine, spermidine, and spermine, their contents in plants under waterlogging stress were recorded. A marked increase in the content of free putrescine, spermidine, spermine by 79.29, 239.13, and 422.69%, respectively in XD20, and by 143.86, 66.67, and 131.36%, respectively in DH662 were observed, compared to CK in response to waterlogging stress (Figure 7A–C). Current results indicate that spermidine application caused an increase in the content of putrescine (25.28%), and spermine (8.68%) in XD20. In contrast, an increase of 58.99, 76.27, and 99.27%, were observed in the content of putrescine, spermidine and spermine, respectively, in DH662 compared to waterlogging alone. Hence, it can be interpreted that exogenous application of spermidine leads to an increase in the content of already present polyamines, thus enhancing the ability of maize plants to cope with waterlogging stress.

### 3.7. Effects of Exogenous Spd on PAs Metabolic Enzyme Activities

**Figure 8:**
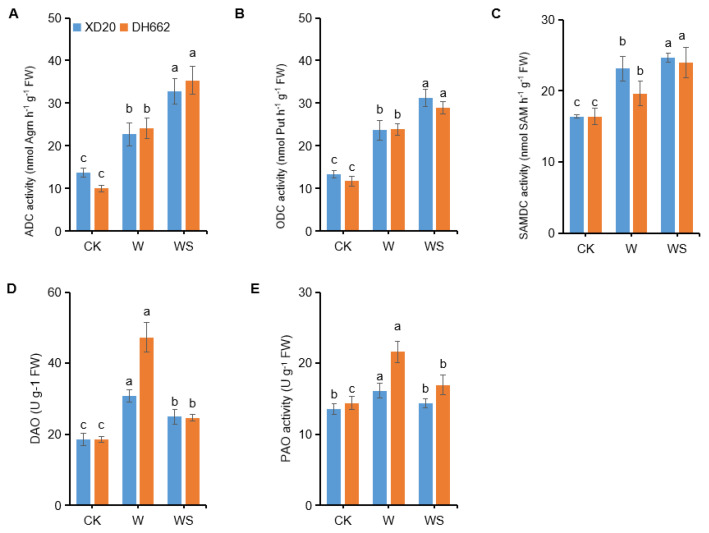
Effects of exogenous spermidine on PA metabolic enzyme activities. ADC (**A**), ODC (**B**), SAMDC (**C**), DAO (**D**), and PAO (**E**). Control (CK) and waterlogged (W) plants were sprayed with distilled water, while WS plants were sprayed with 0.75 mM Spd. Data are expressed as mean ± SD of four replications. Different lower-case letters above bars show marked differences at *p* < 0.05 (LSD test).

PAs play a vital role in plant tolerance against various stresses. PAs are produced in different metabolic processes and are present in all cells. Currently, we tried to investigate the role of PAs by applying spermidine under waterlogging stress and the effects of spermidine on their production level. A significant increase in the activities of ADC, ODC, and SAMDC (65.97%, 77.89%, and 41.08%) was observed in XD20 under waterlogging stress compared to the control, whereas in DH662 an increase of 140.35, 103.42, and 19.67%, respectively was observed (Figure 8A–C). Application of spermidine further led to an increase in the activities of ADC (44.48 and 46.41%), ODC (31.99 and 21.32%) in XD20 and DH662, respectively, compared to waterlogging alone. In XD20 a marked increase in DAO and PAO activities by 65.28 and 19.00%, respectively was observed, whereas an increase in DH662 was 154.70 and 49.87%, respectively compared to the control in response to waterlogging stress (Figure 8D,E). However, compared with waterlogging alone, spermidine application caused a decrease in the activities of DAO (19.08 and 47.82%) and PAO (11.18 and 21.59) in XD20 and DH662, respectively. Thus, it can be concluded that spermidine can enhance PA enzyme activities (ADC, ODC, and SAMDC) and low the activities of PAO and DAO; hence, maize plants become more tolerant to waterlogging stress.

### 3.8. Effects of Exogenous Spd on the Expression of PA-Related Genes

**Figure 9:**
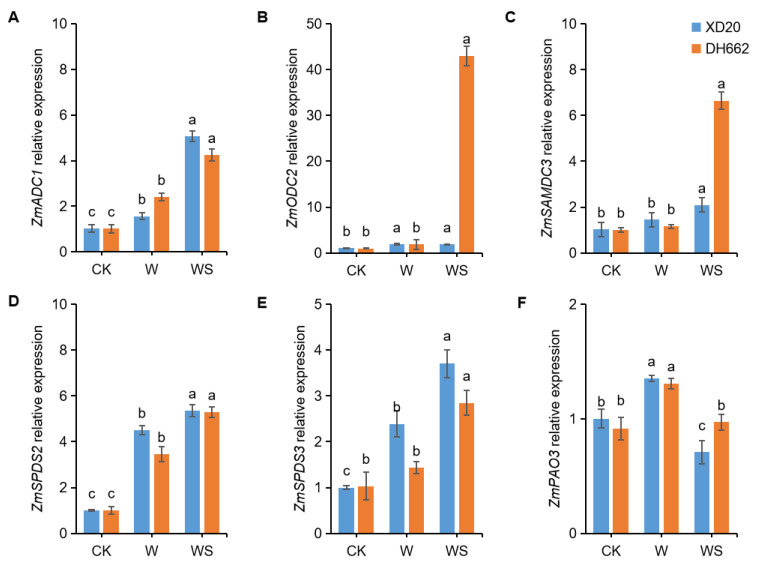
The expression levels of *ZmADC1* (**A**), *ZmODC2* (**B**), *ZmSAMDC3* (**C**), *ZmSPDS2* (**D**), *ZmSPDS3* (**E**), and *ZmPAO3* (**F**), in the leaves of maize under waterlogging stress. Control (CK) and waterlogged (W) plants were sprayed with distilled water, while WS plants were sprayed with 0.75 mM spermidine. Data are expressed as mean ± SD of four replications. Different lower-case letters above bars are pointing to significant differences at *p* < 0.05 (LSD test).

The production or increase in the level of PA-related enzymes is directly regulated by their encoding genes. We examined the expression levels of multiple PA-related genes to gain an overview of the level of tolerance in stressed plants and confirm the role of these genes in initiating defense pathways and inducing plant tolerance against waterlogging stress. The expression levels of *ZmADC1* under waterlogging stress was increased by 53.43% and 138.08% compared to the control in XD20 and DH662, respectively (Figure 9A). Exogenous spermidine application further enhanced the expression of *ZmADC1* under waterlogging treatment, by 222.77% and 76.46% in XD20 and DH662, respectively, compared to waterlogging treatment. The expression levels of *ZmODC2* were increased by 75.14 % and 83.34 % compared to the control in XD20 and DH662, respectively (Figure 9B). Spermidine application significantly enhanced the expression of *ZmODC2* in DH662 by 2244.65%, whereas no momentous change in the expression of *ZmODC2* was observed in XD20. No meaningful change in the expression levels of *ZmSAMDC3* was observed at 3 days after waterlogging. However, exogenous spermidine significantly up-regulated the expression levels of *ZmSAMDC3* compared to waterlogging alone (Figure 9C). Waterlogging induced a significant up-regulation of *ZmSPDS2* by 242.68 % and 351.04 % in XD20 and DH662, respectively, compared to the control. When spermidine was applied exogenously, it resulted in a further up-regulation of *ZmSPDS2* under waterlogging. The expression of *ZmSPDS2* was up-regulated by 53.44% and 18.83% compared to waterlogging in XD20 and DH662, respectively (Figure 9D). The expression levels of *ZmSPDS3* under waterlogging stress was enhanced by 138.78% and 38.93% in XD20 and DH662, respectively, when compared to the control. Exogenous spermidine up-regulated the expression levels of *ZmSPDS3* compared to waterlogging (Figure 9E). Waterlogging down-regulated the expression levels of *ZmPAO3* at 3 days of waterlogging only in DH662 by 61.30%, while no significant effects were observed in XD20. Exogenous spermidine application under waterlogging did not significantly affect the expression of *ZmPAO3* in the two varieties when compared with waterlogging alone (Figure 9F). Overall, these results suggest that spermidine might improve tolerance to waterlogging through regulating PA-related gene expression, thus conferring tolerance to waterlogging in maize plants.

### 3.9. Exogenous Spd Application Down-Regulates Ethylene Biosynthesis

**Figure 10:**
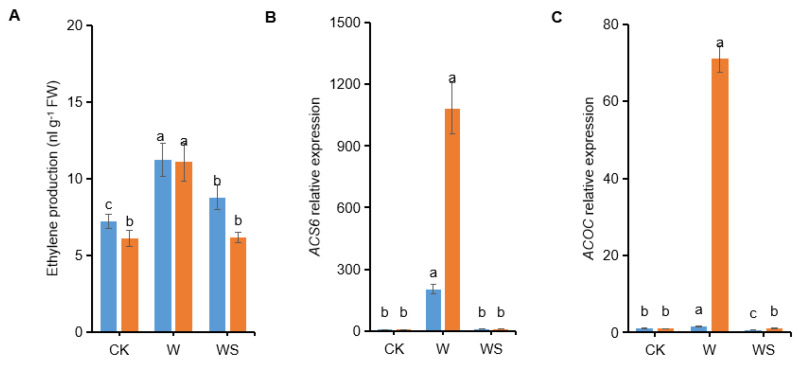
Exogenous spermidine application down-regulates ethylene biosynthesis (**A**) expression of *ZmACS6* (**B**) and *ZmACOC* (**C**) in the leaves of maize under waterlogging stress. Control (CK) and waterlogged (W) plants were sprayed with distilled water, while WS plants were sprayed with 0.75 mM spermidine. Data are expressed as mean ± SD of four replications. Different lower-case letters above bars refer to the significant differences at *p* < 0.05 (LSD test).

Ethylene participates in plant adaptation to flooding, regulating signaling, metabolic responses. Ethylene production increases under flooding stress. Herein, we determined the impact of spermidine application on ethylene production. The leaf ethylene production in XD20 and DH662 increased by 56.09% and 81.51%, respectively in waterlogged plants compared to the control (Figure 10A). However, exogenous spermidine dramatically repressed leaf ethylene production under waterlogging. Leaf ethylene production of XD20 and DH662 in spermidine-treated plants decreased by 21.92% and 44.37%, respectively compared to waterlogging alone after 3 days of treatment. Moreover, the expression of *ZmACS6* dramatically increased by 19,316.76 and 107,219.99% and the expression of *ZmACOC* increased by 61.19 and 6978.94%, respectively, compared to the control (Figure 10B,C). Exogenous spermidine significantly down-regulated the expression levels of *ZmACS6* (98.51 and 99.78%) and *ZmACOC* (58.18 and 98.43%) when compared to waterlogging alone in XD20 and DH662, respectively. Current findings suggest that application of exogenous spermidine might reverse waterlogging-induced ethylene production by suppressing transcription and activity of ACS under waterlogging stress. Hence, it can be concluded that application of spermidine can modify the biosynthesis of ethylene in order to increase the tolerance of maize seedlings to waterlogging stress.

## 4. Discussion

Waterlogging is amongst the key environmental factors limiting plant growth and causes serious damages by affecting crop yields worldwide [32,33]. Tolerance to flooding lasts from a few hours to months depending on the type of plant material being used [34]. Nevertheless, too much water in the soil leads to serious damages to the majority of crops which are not tolerant to waterlogging stress. Maize is among the widely cultivated crops and unfortunately it is also vulnerable to waterlogging stress which poses a serious threat to its productivity in areas receiving heavy rainfall [35].

Waterlogging causes severe damages to plants both phenotypically and physiologically, thus disturbing their growth by inadequate nutrient uptake. These nutritional imbalances along with retardation of root and shoot development leads to chlorosis and then death of the plant [36]. In the current research, it was noted that waterlogging triggered a marked decline in chlorophyll content and growth parameters, but this decline was overcome by exogenous spermidine application (Figure 1). Hence, it is reasonable to conclude that spermidine can improve the negative effects of waterlogging in maize to some extent. Such protective effects of spermidine have already been shown in oats under salt stress [37] and mung beans in low temperatures [38].

Waterlogging exposure frequently leads to the excess accumulation of ROS, thus aggravating oxidative damage to proteins, lipids, and nucleic acids. Inordinate amounts of ROS inside plant cells generate lipid peroxidation while simultaneously increasing O_2_^−^, H_2_O_2,_ and MDA levels. In the present study, the O_2_^−^ production rates, H_2_O_2,_ and MDA accumulation significantly increased in maize under waterlogging stress, whereas exogenous spermidine reduced the rising tendency, which is in agreement with previous studies [39,40]. Antioxidant defense, including SOD, POD, and CAT, usually plays a leading role in preventing damage to plant cell membrane systems as a result of ROS accumulation [41,42,43]. Herein, it was noted that the activities of SOD, POD, and CAT were augmented in response to waterlogging stress in XD20. XD20 scavenged free radicals and ROS in a timelier manner, thus effectively protecting the plasma membrane, and eventually leading to a lower accumulation of membrane lipid peroxidation products (such as MDA) than that of DH662. Furthermore, exogenous spermidine enhanced this activity compared to waterlogging alone. These results indicated that spermidine efficiently reduced ROS accumulation and avoided the degradation of the structure and function of the membrane in maize cells in response to waterlogging. Current results are well supported by earlier reports that observed spermidine efficiently involved in physiologically governing plants upon exposure to abiotic stress [44].

Non-enzymatic antioxidants, such as AsA and GSH, are essential for protecting cells against toxic ROS and keeping the redox balanced under environmental stresses. APX, GR, MDHAR, and DHAR are key enzymes in the AsA–GSH cycle and work together with other antioxidants to scavenge ROS, maintain a balance of ROS, and maintain the stability of cell membranes [45]. APX and DHAR are the two most active key enzymes in the AsA–GSH cycle, and their activities directly affect the content of AsA and DHA. When APX removes H_2_O_2_, it first reduces H_2_O_2_ to H_2_O, thereby removing H_2_O_2_, along with the production of MDHAR. MDHAR is further oxidized to DHA, when MDHAR and DHAR coexist, DHAR uses GSH to reduce DHA, and MDHAR and DHA are regenerated. AsA is generated, and AsA can directly participate in scavenging [46], while GSH is oxidized to GSSG during the reaction process, and then re-reduced by GR oxidation of NADPH. Earlier studies have observed that the primary role of GSH in plant stress tolerance is to promote and maintain a high content of membrane stability. For this reason, GSH and GR levels are believed to be vital signs in plants’ antioxidant status [47]. Meanwhile, the GR activity can directly affect GSH content, and the increase in GR activity is one of the factors that increases GSH content [48]. Studies have shown that APX is the main scavenging enzyme in the cycle, and the rest of the enzymes maintain the balance in the cycle and ensure the scavenging ability of reactive oxygen species [49]. In addition, Kocsy et al. reported earlier that in order to mitigate and enhance the oxidative stress tolerance the AsA/DHA and GSH/GSSG ratios are far more important than AsA or GSH content [50]. Herein, an increase in AsA in the spermidine-treated seedlings grown under waterlogging stress are presumably because of the greater activities of MDHAR and DHAR concomitant to the elevated activity of APX (Figure 4C,D and Figure 5A). GSH content was observed to be increased under stress conditions, and interestingly exogenous spermidine also boosted GSH content (Figure 6A), that could be because of high GSH rejuvenating enzyme (GR) activity (Figure 4B). In the current research it was observed that the exogenous spermidine application leads to an improvement of the AsA/DHA and GSH/GSSG ratios in the maize seedlings (Figure 5D and Figure 6D). Therefore, our findings suggest that spermidine may well improve the antioxidant capacity by adjusting the non-enzymatic antioxidant system as well as maintaining redox balance [51]. Moreover, recycling of ascorbate–glutathione by applying spermidine could accelerate plant tolerance towards abiotic stresses. Altogether, current the study supports the assumption that spermidine-treated plants, under waterlogging stress possess a higher ability to activate ROS scavenging mechanisms.

Due to their positive charge at physiological pH, PAs are implicated in the procurement of plant tolerance against multiple stresses [52]. It has been confirmed that PAs are assume an inherent structural role in the binding and alleviation of negative macromolecules, such as DNA, RNA, proteins, and phospholipids. It is reported that a higher (spermidine + spermine)/Put ratio is beneficial for plants to offset abiotic stresses [37]. However, in the current study, a rise in the PAs levels was noted in response to waterlogging, among which Put in the sensitive DH662 had a much higher increase than tolerant XD20, which indicated that waterlogging caused excessive accumulation of Put. Accumulation of Put under waterlogging stress may be a stress response rather than a protective mechanism. Compared with waterlogging alone, spraying spermidine further increased PA levels, and the boost of spermidine and spermidine was greater in DH662 than in XD20. This suggests that spermidine could act to maintain high spermidine and spermine levels during stress. Therefore, in this study, spermidine was used to examine the protective mechanism of PA in maize seedlings during waterlogging.

Prior studies have shown that spermidine stimulates PA metabolic enzyme activity, therefore affecting endogenous PA concentrations during freezing [53]. In plants, decarboxylase reactions which is catalyzed via ADC or ODC results in the formation of putrescine from arginine. Additionally, spermidine and spermine are produced from Put in subsequent reactions by adding aminopropyl moieties resulting from decarboxylation of S-adenosylmethionine which is catalyzed through SAMDC. Putrescine deprivation is in line with DAO, although spermidine and spermine degradations are catalyzed by the PAO. Findings suggest that gene expression levels along with PA synthesis enzymes ADC, ODC, SAMDC, together with catabolic enzymes such as DAO, and PAO, also dramatically rose under waterlogging, thus suggesting that PA metabolism was improved by waterlogging (Figure 8). The higher increase in PAO activity and gene expression might cause lower spermidine and spermine levels in the sensitive DH662. Furthermore, we observed that in waterlogging conditions the application of spermidine led to an increase in the activities and gene expression levels of enzymes such as ADC, ODC, and SAMDC, even though it lowered DAO and PAO enzyme activities and gene expression levels in maize plants. In waterlogging conditions spermidine application resulted in the up-regulation of SPMS and SPDS genes meanwhile lowering the PAO and DAO activities, thus maintaining the higher levels of spermine and spermidine to mitigate stress by increasing PA levels. Current findings suggests that spermidine leads to an improvement in waterlogging tolerance by regulating PA metabolic activity under transcriptional and translational control in maize plants.

Involvement of PAs in plants tolerance to numerous environmental stresses has been well detailed [54,55]. In the current research a decrease in photosynthetic activity along with leaf injury symptoms, such as wilting and chlorosis were observed as reported earlier [56] (Figure 1). The appearance of symptoms due to waterlogging is a sign of ethylene production as well as various other limiting factors [57]. In the current analysis the production of ethylene in maize seedlings was enhanced with the increase in waterlogging stress as reported earlier in perennial pepper weeds [58], avocado, and cotton [11] causing leaf senescence [11]. Ethylene exacerbates the impacts of abiotic stresses, whereas the harmful effect of waterlogging on plants can be mitigated by lowering the levels of endogenous ethylene in plants [59]. Herein, a significant decrease in ethylene production in maize plants was observed in the plants that were pre-treated with spermidine in waterlogging conditions, showing the importance of spermidine application in mitigating stress tolerance (Figure 10A).

**Figure 11:**
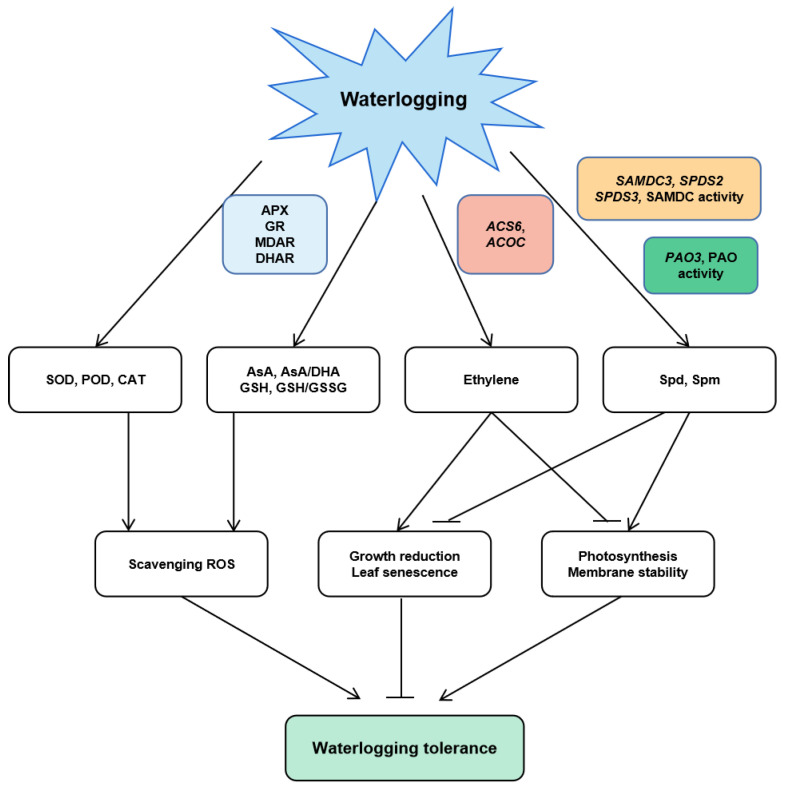
Suggested pathway for spermidine-mediated waterlogging stress response in maize derived from the results involving the antioxidant system, ethylene, and PA metabolism. “
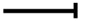
” points to inhibition and “
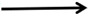
” points to activation.

ACS and ACO during the process of ethylene biosynthesis are governed by different exterior and interior factors for controlling ethylene production. Higher ACC production in the roots is then conveyed to the shoots of the plant, in which it is changed to ethylene by ACO [60]. Herein, waterlogging conditions led to an elevated expression of *ACS6* and *ACOC* in maize plants along with increased ethylene production. At the same time, the spermidine application repressed *ACS6* and *ACOC* expressions, accompanied by a decrease in ethylene production (Figure 10B,C). These findings recommend waterlogging stress mitigation through exogenous spermidine application in maize linked to ethylene biosynthesis at the transcription level. The proposed mechanism of spermidine-facilitated response to waterlogging in maize is shown in Figure 11.

## 5. Conclusions

Maize plants subjected to waterlogging presented an inhibition of growth. Two maize varieties (a waterlogging-tolerant variety—XD20 and a waterlogging-sensitive variety—DH662) were subjected to waterlogging stress at the seedling stage, and then foliar was sprayed with 0.75 mM spermidine. Lower chlorophyll content and reduced growth indices were observed in the two maize varieties under waterlogging stress compared to the control treatment. Similarly, we observed a considerable increase in the superoxide anion (O_2_^−^) generation rate, and H_2_O_2_/MDA accumulation in the two maize varieties under waterlogging stress compared to the control treatment. Foliar application of spermidine significantly increased antioxidant enzyme activity under waterlogging stress. On the other hand, spermidine application enhanced tolerance to waterlogging by contributing to reducing the excessive accumulation of ROS and maintaining plant growth. This could be related to spermidine enhancing the capacity of the antioxidant system. Likewise, spermidine causes an increase in PA levels under waterlogging stress and reduced ethylene production. Consequences of spermidine application were most apparent for the waterlogging-sensitive cultivar DH662 under waterlogging than the waterlogging-tolerant variety XD20. Briefly, the results suggest that spermidine increases the tolerance of maize plants to waterlogging by enhancing the antioxidative capacity and modulating polyamine and ethylene biosynthesis. This experiment delivers key scientific information to deeply understand the mechanisms of spermidine in promoting maize waterlogging tolerance. However, more research is required to deepen our understanding of the molecular and genetic mechanisms involved in waterlogging tolerance in maize.

## Data Availability

All data used in this manuscript are available within the text and its Supplementary Materials.

## Supplementary Materials

The following supporting information can be downloaded at: https://www.mdpi.com/article/10.3390/life12111921/s1, Table S1: Results of normality test and homogeneity test of variance. Table S2: List of Primers used in this study for qRT-PCR.
